# Supplementary material for: Complexome profiling on the Chlamydomonas lpa2 mutant reveals insights into PSII biogenesis and new PSII associated proteins
Source: J Exp Bot. 2021 Aug 26;73(1):245–62. doi: 10.1093/jxb/erab390 (PMC8730698; doi:10.1093/jxb/erab390)
Supplement: erab390_suppl_Supplementary_Dataset_S1 [file erab390_suppl_supplementary_dataset_s1.zip › Supplemental Dataset 1 - Excel List and all profiles/plots/NDA1_Cre16.g691552.html]

Trivial name: NDA1  
  
Euclidean distance: 26117.68  
Mean Intensity (WT): 1347.63  
Mean Intensity (Mut): 257.31  
Distance: 19.38  
  
MapMan: mitochondrial electron transport / ATP synthesis.NADH-DH (type II).external;mitochondrial electron transport / ATP synthesis.NADH-DH.type II.internal matrix;mitochondrial electron transport / ATP synthesis.NADH-DH (type II).internal matrix  
  
p value of intensity sums Welch test: 0.2247
